# Presumption of Survivorship

**Published:** 1843-06

**Authors:** 


*Presumption of Survivorship.*—(Continued from page 352.)

*Thirdly.* When many persons perish at the same time by drowning.

The following circumstances are to be taken notice of in cases of this kind:

1. Persons, who are able to hold their breath a long time, will die later than those who cannot. Col. Townshend placed himself in the presence of many physicians in a state resembling death. He laid himself upon his back, one physician felt his pulse, another beating of the heart, the third held a mirror before his mouth. Pulsation and beating of the heart ceased instantly, the mirror remained clear, this state continued for half an hour, when the beating of the heart and respiration returned. (*Henke's Zeitschrift*, 4 *Jahrgang*).

2. Persons who fall head foremost into the water, will die earlier than those who fall in another manner.

3. Persons who remain longer upon the surface of the water, or several times rise up again, will die later than those who sink immediately to the bottom.

Dissection of such drowned persons affords the principal grounds of decision, whether they have lost their lives by apoplexy or suffocation.

Apoplexy causes death sooner than suffocation. But should many persons have died of apoplexy or suffocation, we may certainly assume, that the persons with whom a tendency to apoplexy prevailed, have died sooner than those where this was not the case. With persons inclined to apoplexy, death on falling into the water, particularly if they are heated, occurs instantly." (*Henke's Lehrbuch*, § 473).

But where many persons have died at the same time of suffocation, we may assume, that the persons on whom the signs of suffocation are the most distinct and marked, have died last.

A body, on which the signs of suffocation and apoplexy are found at the same time, has died earlier than one on which we find merely the signs of suffocation.

A lady of noble birth, E. M. v. L. was passing with her three daughters, of 14, 16, and 18 years of age, and her cook in a two horse-coach through the ford at Pradel, which was very much swollen: the king-bolt of the carriage came out and the horses drove on with the fore part of the carriage. The back part sank down, and was carried somewhat forward by the water, whereupon the coachman springs from his seat, in order to assist the persons sitting in the carriage, but which was impossible, as the water in the stream carried the carriage across and overturned it, which he did not let go of, and thought to keep hold of, which however was impossible, because he, at the time when the carriage was turned over, was turned over with it. When the carriage had been turned round some three times, they were all still alive; but when the carriage rose up the fourth time, the three young ladies were already dead, the lady and the cook were still alive, while the first three were fallen down, and did not move, and had also partly fallen out backwards: the cook had still cried out, and the lady said to him, Oh! coachman save yourself only; as he had then at three different times been drawn by the current under the water. When the water had again turned over the carriage, the lady yet cried out and prayed; but after that the coachman had seen no one again, and was himself forced on by the current, until he came to a tree, which he seized.

When now the maternal relations claimed the whole inheritance, to the exclusion of the paternal relations, the latter sought to invalidate the testimony of the coachman, and based themselves upon the presumption of law, that the children might have lived longer than the mother, and if not all, yet at least one had survived the mother. Upon this very doubtful case the following questions were propounded.

*a.* Whether from all the occurring circumstances, that, what the coachman has testified of the first ensuing death of the three daughters, deserved credit, and that therefore it was to be held probable that the mother survived the daughters.

This question was thus answered: although *ex actis* it does not appear, who had drawn the drowned bodies from the water, how soon it was done, whether any one was near by, and had traced motion, respiration, warmth, or any signs of life, on any of the bodies; also that the fishermen, who were in the neighborhood, or any others had been examined; further, the coachman has not explained what he meant by springing from the seat, how, and where he had stood, that he possibly would have held on the wagon, and had perceived what had taken place therein, as he has related it: besides we do not find how old the drowned lady Von L. was, hence we had to investigate the point. But since the answer to the proposed question, first concerns that, what the coachman could perhaps immediately perceive, and judge of according to the circumstances and also to the appearances, but especially to the others, how such things might deceive, and he not be able to know the real death of the three ladies; yet from the legal documents we may believe, that the bodies were drawn out from the water as soon as possible, that no sign of it was observed in either, while an evidence of that kind might have decided the whole question, and it is well remarked, that the coachman, at the time he observed what was taking place with and in the coach, had his head above the water, also from the age of the young ladies, the age of the mother was about 40, more or less.

Hence in reference to the first point of the answer: it is so, that in the anguish and danger of death, wherein the coachman found himself, he could with difficulty observe the course of things, and it is not indeed possible, that he from the falling down, and want of motion, at such a moment could correctly decide, whether the ladies, especially three of them, together with the mother and the cook who were in the coach, were really dead, still from the testimony of the coachman, that the lady had spoken the last, and on the fourth turning of the carriage the daughter had fallen down, and did not move; we hold it probable, that in so far as it can appear from the coachman, it must be, that the three ladies had died before the mother.

But this account of the evidences of death, uncertain and founded only on conjecture, was still further opposed by another question, in which the reasons *pro* and *con* were decisive.

6. Whether, and how far, despite the testimony of the coachman, still the ladies may have lived, when he believed that they were already dead?

*a.* When then on the side of the maternal relatives in the present case, it was maintained (*a*) that the mother, of a grave disposition, on account of her age, and ever had been so, consequently at the time was well, hence in so sudden a necessity, had more determination and energy than the three young ladies, and could help herself for a longer time, as also the testimony of the coachman conforms hereto, and strengthens it the more, (*b*) also that we do not find, as with the bodies drawn out from the water any signs of life remaining was discovered, (*c*) as also such extraction or drawing forth of bodies of any kind, would have occasioned irritation and motion, if, indeed, with one or the other, any had been left: (*d*) in addition, the delicate young ladies by excitement would have been brought rather to cramps in the breast and convulsions, and hence death would be hastened, so that the mother survived them, (*e*) consequently the lying down and want of motion in the young ladies, which the coachman testified to as the signs of death, are also on this account the more sufficient to prove their death, and hence to confirm the testimony of the coachman and the perfect death of the daughters before the mother, therefore the maternal relatives are entitled to maintain the inheritance of the daughters descended to the mother who died last, to the exclusion of the relatives of the father.

*b.* On the other hand the relatives of the father contended that (*a*) the vital power at the age of the three ladies was generally able longer to withstand death, than at the age of the mother, as the latter had been affected with hemorrhage and other ailments, and (*b*) that after the bodies were drawn out, no bleeding, warming or rubbing, &c., was made use of, as these attempts might be useful to restore life or as a possible proof of the death, as (*c*) the motion of the bodies at the time of the drawing them out from the water and the coach, is in no manner to be compared with the carrying of them, and (*d*) farther syncope and cramps in the breast in which the three ladies had earlier fallen, so much the less proves death, as it is well known, that, particularly with females, in the like cases, besides that they grow faint and numb, frequently the pulse cannot be felt in the external arteries, as also respiration without a movement of the breast, and only by a slight motion of the diaphragm be discovered, and yet such persons recover, hence (*e*) we particularly cannot know, whether and how long the young still had life, they might have swam away, and the stream have carried them on more alive than absolutely dead: besides also the lying down and immovableness of the three ladies, which the coachman testifies to, still less, than the outcry of the mother, as they were found in the water, can prove the absolute death of all these persons; the rather (*f*) that these and many more signs may deceive, although (*g*) people, who have been a considerable time under the water, indeed often recover, if application is made use of; as the young ladies were three in number, and on the 3d of September, the water was not so very cold, it is the more evident, that when they were brought to the bank, if not all three, together with the mother, yet one had a spark of life and might have been saved. For these and other reasons, together with the presumption of law, we consider that the testimony of the coachman deserves no credit.

*Fourthly.* When many persons are precipitated into an abyss and dashed to pieces.

In this case we must particularly examine, on whom wounds of the nobler organs are found. Thus, for example, those will die earlier whose skull is fractured, than those who have suffered the fracture of an arm or leg. Those also will have died the earliest, on whose corpses we find the internal vessels lacerated, than those who have received a flesh (bleeding) wound on the extremities. But where many persons in the same manner, and by the same injury are killed, he will have died the earliest on whom the injury first happened, or also that one on whom the severest lacerations are found. But where the wound is equally great on many persons, or has happened to all on one and the same organ, then the stronger will have survived the weaker. The idiosnycrasy of the person may, however, in the last case, afford a ground of distinction, as when, for example, death is caused by extravasation of blood in the skull, a full-blooded person or one who has a tendency to apoplexia sanguinea, will die sooner than those with whom this is not the case.

*Fifthly.* When many persons perish at the same time in a conflagration.

In such a case we must first ascertain, whether the persons are really burnt, or suffocated by smoke, or entombed and crushed by the falling buildings.

The distinctive signs of persons dead from suffocation and wounds have already been mentioned.

In respect to death by burning we must decide, whether the man was really burned alive, or whether we find signs upon the dead body, that the marks of burning have been occasioned after death, resulting from some other cause. On this point we have a very interesting essay by Christison.

A case of many persons destroyed by fire, which on account of the claims set up to the inheritance occasioned judicial proceedings, is to be found in Kopp's Jahrbuch der Staatsarzneikunde, 71, 8, p. 181. At Ormay, in the district of Murten in the Canton of Fryburgh, a fire in the night of the 16th and 17th of June, 1809, broke out in the dwelling of a countryman, Jean Etter. The man was absent, and expected to return the same evening, his wife on that account had remained, according to her custom, waiting for him in the room, where she was found alone with her infant. The other four children slept in a little adjoining chamber. All the five children together with their mother perished in the fire.

It was impossible to discover in what part of the house the fire originated. The body of the mother was found nearly destroyed by the flames. Her infant, with its body almost uninjured, lay under her. A few steps further lay the oldest daughter, 14 years of age. The three other children were found together in the centre of the hall.

There was no marriage contract, and the father remained for a while in the quiet possession of the inheritance of his wife, who had brought to him on the marriage a considerable real estate from her paternal and maternal side, as also some stocks and money.

The provincial law in force there permitted to the surviving husband, the usufruct for life in the property of the wife; but, when he claimed the inheritance, he must present an inventory and give security. According to the laws the property of intestates belonged to the nearest blood relations, who have survived the deceased.

When Etter was about re-marrying, the sister of the deceased claimed, that he should fulfil the requirements of the law, but he refused and the court decided, that the inheritance should be divided: but with this the sister was not content, and her advocate appealed from the provincial law, since, because it was not proved, whether the mother or her children had survived, it must be assumed according to the Roman law and the code Napoleon that the mother was the survivor, and consequently that the sister was the heir of the deceased.

On the other hand, the counsel for Etter contended, on the same authorities, that it was not sufficient, that Marie Knoff for the establishment of her claim, should represent herself as the nearest relative of the deceased, but it rested solely on this, whether she at the time of the death was her next blood-relation, which could not be the case, if only one of the five children had died after the mother. He attempted to prove, how extremely improbable it was, that the mother had survived all the five children, and produced the following striking circumstances.

1. The children, who perished with their mother, were five, and their bodies were found in different parts of the room; they could not thus all at the same time, in the same moment have lost their lives, as was before assumed, if for example, they had all been entombed under the ruins. Also in reference to their different positions it is almost a certainty, that one or another of the children had lived longer than the rest, and it is the extremest improbability, that all five of the children should have died before the mother, and not even one, were it only for a second, had survived her. As to the survivorship of one of the children we may place five against one.

2. The body of the mother was so much destroyed by the flames, that it excited horror, while that of the child, which she had under her. was scarcely injured. The unhappy mother had used her best endeavors to protect her child, and forgot on that account her own danger. Without doubt she covered it with her body and her dress, it was thus covered from the flames and less exposed to the effect of the smoke, while the mother was entirely exposed to them. We have thus reason to assume the survivorship of the child, in respect to the temporary (*momentanen*) protection derived from the mother.

3. The oldest daughter, about 14 years of age, was found a few steps from her mother, her body was entirely uninjured, so that her hair and the ribbon upon it exhibited not the slightest trace of burning. This proves, that not the flames, but merely suffocation occasioned her death. We must not forget, that she slept in the little adjoining chamber, and that, when she came out, the firesmoke must have already reached her mother, even if the flames had not yet destroyed her. At least the following circumstance gives strong reason to conclude, that she was already dead and had perished by the flames and smoke, when the children left their chamber.

4. Three of these children were found together, in the centre of the hall. They had thus fled from the chamber, come through the common room, had opened that door and thus reached the front door. Could they have opened the front door, they would have been saved. Hence it is in the highest degree probable, that already on their flight the mother was dead, for it is natural, that if she had been still alive, she would also have attempted an escape, and have aided all, to reach the front door.

The decision of the superior court is unknown to the author.

A fire broke out at night in the year 1546 in the Castle Blankenburg on the Hartz, and the count and his pregnant wife, as well as the steward and his wife could not ecsape from the flames. They escaped from one chamber to another, until the two females were suffocated by the smoke; the two men remained alive and were at length taken out from the flames. The countess was 37 years old, the age of the other persons is not given.—*S. Thïeringen und der Harz.* 81 *Heft.* page 56. *Sondenhausen bei Eupel* 1840.

*Sixthly.* When many persons die at the same time of poisoning.

In the poisonings of many persons by one and the same kind of poison we consider those as dead first, on whose bodies we find the greatest destruction by the poison.

On this point no decision can be based, since indeed the greater the degree of the destruction in the body presupposes a longer life, and there are poisonous substances which produce no destruction in the body, and in corrosive poisons arsenic acts in a twofold manner, as it kills by an effect upon the nervous system, or occasions an inflammation of the stomach and intestines passing into gangrene, to which in the decision of such a case reference must be had, Even in the case, where a person poisons another and afterwards poisons himself, the knowledge of the person of the mixer of the poison is not sufficient to decide the priority of death, since if the poison administered was arsenic, the poisoned person, who first had taken the poison, still may have died last, if the poison produced its effects more slowly upon him, while the poisoner may have died earlier than the former by the more speedy effect of the poison.

Death by poisoning appears to proceed in the reverse order of death by hunger or by bleeding; and the elder to die before the younger.

Persons may die at the same time apparently by poisoning, without this really being the case.

Dr. Roloff relates the case of two men who died suddenly and probably by poison, with whom on dissection no trace of poison could be proved, although the symptoms of disease, as also the appearance on dissection indicated a poisoning, so that Roloff had to leave it undecided, whether the two young people had died of gastritis, enteritis, cholera or malignant typhus. They were two brothers, of whom the eldest was 18 and the youngest 13 years old; the first died Feb. 3, and the last Feb. 21.—*Kopp. Jahrbuch des Staatsarzneikunde,* 7 *Jahrgang* 1814.

*Seventhly.* When many persons die at the same time of wounds.

Klose lays down in this case the following principles:

1. With duellists, who die at the same time, the degree of mortality decides for the earlier or later death. 2. If from an examination of the bodies it appears, that the one had been murdered by another, and the murderer had then killed himself: Klose assumes, that the suicide had died last, and cites a recent case, where a man murdered his wife with an axe, but drowned himself. In this case the husband had survived the wife. But on the other hand, had the man thrown the wife in the water and she had died of suffocation, while he had shot himself, the wife may very easily have survived the husband.

3. When in cases of wounds of the same kind the wounded organs of a person are morbid or weak, we may assume, that the persons, who are of the weaker organisation, have died first.

4. If a cannon ball strikes several persons we may assume, that those have died first, who were struck first.

In the siege of Breslau, a soldier was struck by a cannon-ball on the shoulders and was killed on the spot; the same ball crushed the head of another soldier standing several hundred feet distant.—*Klose, System*, p. 395.

With persons of different sexes, but with wounds of the same nature, Klose assumes, that the female will have died earlier than the male. But it is sufficiently known, that bleeding with women is less dangerous and less speedily prejudicial, and we must therefore assume that the man in such a case has died earlier than the woman. Besides, nature stifles in woman bleeding sooner than with men, since the former are inclined to fainting, which is the best means of stopping bleeding.—*Klose, über der Einfluss des Geschlechtsunterschilds*, p. 246.

*Eighthly.* When many persons die at the same time by cold.

*Bernt*, *Henke* and *Mendel* assume apoplexia cerebralis as the only cause of death from cold; but Niemand (*Handb. der Staatsarzneikunde*, p. 210,) and Granland (*Dissertat. de Asphyxia congelatorum*, Helsingforsiæ 1832) place the cause of death in asphyxia. Metzger assumes apoplexy as the cause of death; but says that cold with a low but uninterrupted degree of coldness, occasions death by its fatal effects upon the nervous system and the heat of the body; death by cold may be thus sudden or protracted. With these opinions the results of dissection of those who have died from cold coincide. In general, we find in these cases the skin uncommonly pale, the vessels of the head empty, the intestines very full of blood, and the lungs near the pleura inflamed. Klose, l. c. p. 429. Cappel (*Acta Natur. Curiosorum*, vol. 3, obs. 28,) found congestion in the breast and the abdomen, and Plouquet (*von gewalsamen Todesarten*) found the vessels of the head and the lungs filled with blood.

The prevalent circumstances, whether a person was stronger, fatter, better clothed than another, whether one had a tendency to apoplexy or fainting or had drunk ardent spirits, must in reference to the appearances on dissection afford an evidence as to the earlier or later death of the one person or the other. Children and old people are killed by cold, more easily than persons in middle age: but women, who are in the climacteric years, are killed by cold under the same circumstances not so easily as men, because they possess proportionally a warmer blood and more animal heat (warmth of body).

*Ninthly.* When many persons perish at the same time of hunger.

When persons die from hunger at the same time, the place, where it has happened, must first be considered. Thus, death follows later with those who are found in a damp place, a cellar, &c., where they can breathe a moist air or can drink. In reference to sex, women can endure hunger longer than men, who require a greater quantity of more nourishing and stimulating food. Further, weak persons will perish from hunger sooner than stronger ones, younger persons than older ones. Young active men will be destroyed by hunger sooner and more severely, than phlegmatic, quiet, less respirable, old persons, who require less food, than younger ones.

The truth of this position is proved by the melancholy end of Count Ugolino in Pisa, whom the citizens together with his family, closed up and starved in the year 1283, in the Hunger-castle so called. The youngest child, a boy of 3 years old, died first on the fourth day, the three others in youth, the fifth and sixth day, the father in the prime of life, died the eighth day. Dominicus Sala, *Tractat. de alimentis*, sect. 1, p. 32. In Oppido, a maiden lived eleven days under the ruins, and in the most horrible proximity to a corpse. She was 15 years old, and the attendant of a child. On the falling of the house, she held the child firmly in her arms, who, tormented by the most torturing thirst, died on the fifth day. Till then she had preserved her presence of mind, but afterwards she experienced the most painful sensations of hunger and thirst. Her despair passed into perfect senselessness, and at the time she felt nothing of the pain of the dislocation of her hip, on the falling of the house. Drink was the only thing which she desired after her deliverance. When asked respecting her condition under the ruins, she answered: *I slept.* Many of those entombed persons were found on being dug out in a stupor-like sleep, in which they had sunk by the falling or after some days, according as their nervous system was strong or weak; many considered themselves as intoxicated.

An old woman lay seven days under the ruins in a state resembling sleep; when she awoke, her only complaint was of burning thirst.—*Amer. Journ. Med. Scien.*, from *Wildberg's Jahrbuch der Gesammtén Staatsarzneikunde.*

